# Association between maternal mental health, the COVID-19 pandemic, and children’s developmental outcomes in Scotland

**DOI:** 10.1186/s13690-025-01572-w

**Published:** 2025-03-27

**Authors:** Kenneth Okelo, Louise Marryat, Aja Murray, Josiah King, Iain Hardie, James P. Boardman, Michael V. Lombardo, Sarah Stock, Rachael Wood, Bonnie Auyeung

**Affiliations:** 1 Department of Psychology, School of Philosophy, Psychology and Language Sciences, University of Edinburgh, 7 George Square, Edinburgh, EH8 9JZ UK; 2 School of Health Sciences, University of Dundee, Dundee, UK; 3 Centre for Clinical Brain Sciences, University of Edinburgh, Edinburgh, UK; 4 Centre for Reproductive Health, Institute for Regeneration and Repair, University of Edinburgh, Edinburgh, UK; 5 Laboratory for Autism and Neurodevelopmental Disorders, Center for Neuroscience and Cognitive Systems, Istituto Italiano di Tecnologia, Revereto, Italy; 6 Usher Institute, University of Edinburgh, Edinburgh, UK; 7 Public Health Scotland, Edinburgh, UK

**Keywords:** Birth during COVID-19, COVID-19 pandemic, Mental health difficulties, Mental illness, Child development, Developmental delays

## Abstract

**Background:**

The number of reported maternal mental health (MH) difficulties during the COVID-19 pandemic was higher than during the pre-pandemic period. Findings on the link between the COVID-19 pandemic and children’s developmental outcomes suggest lower scores on the Ages and Stages Questionnaire (ASQ-3) among children born during the COVID-19 pandemic compared to pre-pandemic cohorts. The present study explored the interaction between maternal MH and being born during the COVID-19 pandemic on children’s developmental outcomes. Furthermore, it examined the combined effect of maternal MH and birth during the pandemic on children’s developmental outcomes.

**Study design:**

This study used a linked administrative dataset from Scotland. Children born between 1st March 2020 and 30th June 2021, inclusive (*n* = 32,683), and a comparative historical cohort that included those born between 1st April 2017 and 31st October 2018 in Scotland (*n* = 50,257) were included. Regression models were used to adjust for covariates, with outcomes such as ASQ-3 scores and developmental concerns and predictors such as maternal MH and birth during the COVID-19 pandemic.

**Results:**

A history of MH hospital admission was associated with increased odds of developmental concerns: OR = 1.038, 95% CI [1.012, 1.064], *p* = 0.004** and reduced ASQ-3 scores (effect size = 0.130, 95% CI [-0.204, -0.056], *p* < 0.001***). There were mixed findings on the association between being born during the COVID-19 pandemic (developmental concerns: OR = 1.024, 95% CI [1.019, 1.029], *p* < 0.001***) and ASQ-3 scores (ES = 0.012, 95% CI [-0.002, 0.025], *p* = 0.08) but no interaction between a history of MH hospital admission and birth during the COVID-19 pandemic. However, there was an interaction effect on mental health assessed by psychiatric outpatient attendance records association and birth during the COVID-19 pandemic on the ASQ-3 scores SD; -0.07 (ES =-0.066, 95% CI [-0.106, -0.027], *p* < 0.001***).

**Conclusions:**

Our findings suggest that being born during the COVID-19 pandemic and maternal MH influenced child development with relatively small effects, with mixed findings on their combined presence. Our study only examined developmental outcomes up to age 13–15 months. Future studies should explore the potential long-term effects of being born during the pandemic and MH.

**Supplementary information:**

The online version contains supplementary material available at 10.1186/s13690-025-01572-w.

**Table:** 

**Text box 1. Contributions to the literature**
• There is an association between being born during the pandemic and maternal mental health and children’s developmental outcomes, respectively
• Studies have investigated how being born during the COVID-19 pandemic interacts with maternal mental health to affect birth outcomes. Yet, little focus has been on children’s developmental outcomes after delivery
• This study provides new insights into the impact of maternal mental health on children’s developmental outcomes within the context of the COVID-19 pandemic, utilising a large administrative dataset
• This may support public health researchers, policymakers, and practitioners in detecting and treating individuals with MH problems and targeting support for the offspring of mothers with MH diagnoses

## Introduction

Maternal mental health during pregnancy and postnatally has been linked to children’s developmental outcomes, especially behavioural outcomes [1–3]. Postpartum depression, for example, has been linked to behavioural problems in infants and poor maternal-infant attachment. Children with behavioural problems often have a greater risk of poor academic performance and lower school readiness [4, 5]. In addition, this might affect other domains/areas of growth and development, such as the child’s health. Previous work has shown that maternal stress affects offspring’s cognitive development, negative affectivity, neurodevelopment, psychiatric disorders, and difficult temperament [6].

Maternal mental health difficulties during the COVID-19 pandemic were on the rise compared to the pre-pandemic period [7, 8]. Studies have observed greater levels of mild depression among individuals who gave birth during the COVID-19 period than among those who gave birth during the pre-pandemic period [7, 9]. Pregnant individuals were particularly vulnerable to ‘pandemic-related stressors’ due to disruptions in antenatal care services, the possibility of contracting COV, financial concerns, and reduced physical activities [10–12]. In addition, Public Health and Social Measures (PHSM) during the COVID-19 period, such as social distancing and working from home (where possible), also led to job losses [13] and an increase in the number of reported maternal mental health difficulties [14]. This rise in mental health difficulties could also be attributed to the reduction in protective factors such as access to social support, health services and income stability [15–19].

Similar to the impact on pregnant individuals, children were impacted by the pandemic in terms of a lack of social support and reduced opportunities for in-person activities [20]. In a study in the USA, parents reported that children aged 0–5 years missed out on social development opportunities [21]. On their developmental outcomes, those born during the pandemic showed a higher likelihood of developmental delays as they had lower scores on the gross motor, fine motor, communication, and personal-social sub-domains of ASQ-3 scores compared to the pre-COVID-19 pandemic cohorts [22].

There is evidence of the associations between being born during the pandemic and maternal MH and children’s developmental outcomes, respectively. However, to our knowledge, no research has been conducted on the interaction effect of being born during the pandemic and maternal MH on children’s developmental outcomes. Noting that maternal MH protective factors such as social support and childcare access were not consistently available during the COVID-19 pandemic, it is important to understand the potential interaction effect of maternal MH and the COVID-19 pandemic on children’s developmental outcomes. This study used the population-based linked administrative health dataset covering all children born in Scotland between 1st April 2017 and 30th June 2021 who had developmental outcomes assessed at routine 13–15 month child health reviews.

Specifically, we address the following questions:

1. Is there an association between maternal MH (having a history of MH hospital admission/psychiatric outpatient attendance) and children’s developmental outcomes measured by the Ages and Stages Questionnaire (ASQ-3) assessment scores in both cohorts (pre-COVID-19 cohort and COVID-19 cohort) at age 13–15 months?
2. Is there an association between maternal MH (having a history of MH hospital admission/psychiatric outpatient attendance) and child developmental concerns identified by health visitors during early child health reviews in both cohorts (pre-COVID-19 and COVID-19)?
3. Are there interaction effects of the COVID-19 pandemic and maternal MH (having a history of MH hospital admission/psychiatric outpatient attendance) on children’s developmental outcomes measured by the (ASQ-3) assessment scores?
4. Are there interaction effects of the COVID-19 pandemic and maternal MH (having a history of MH hospital admission/psychiatric outpatient attendance) on child developmental concerns identified by health visitors during early child health reviews?

## Methods

### Design

This study used administrative datasets in Scotland to compare the association between maternal MH and children’s developmental outcomes before and during the COVID-19 pandemic.

### Data and participants

**Fig. 1:**
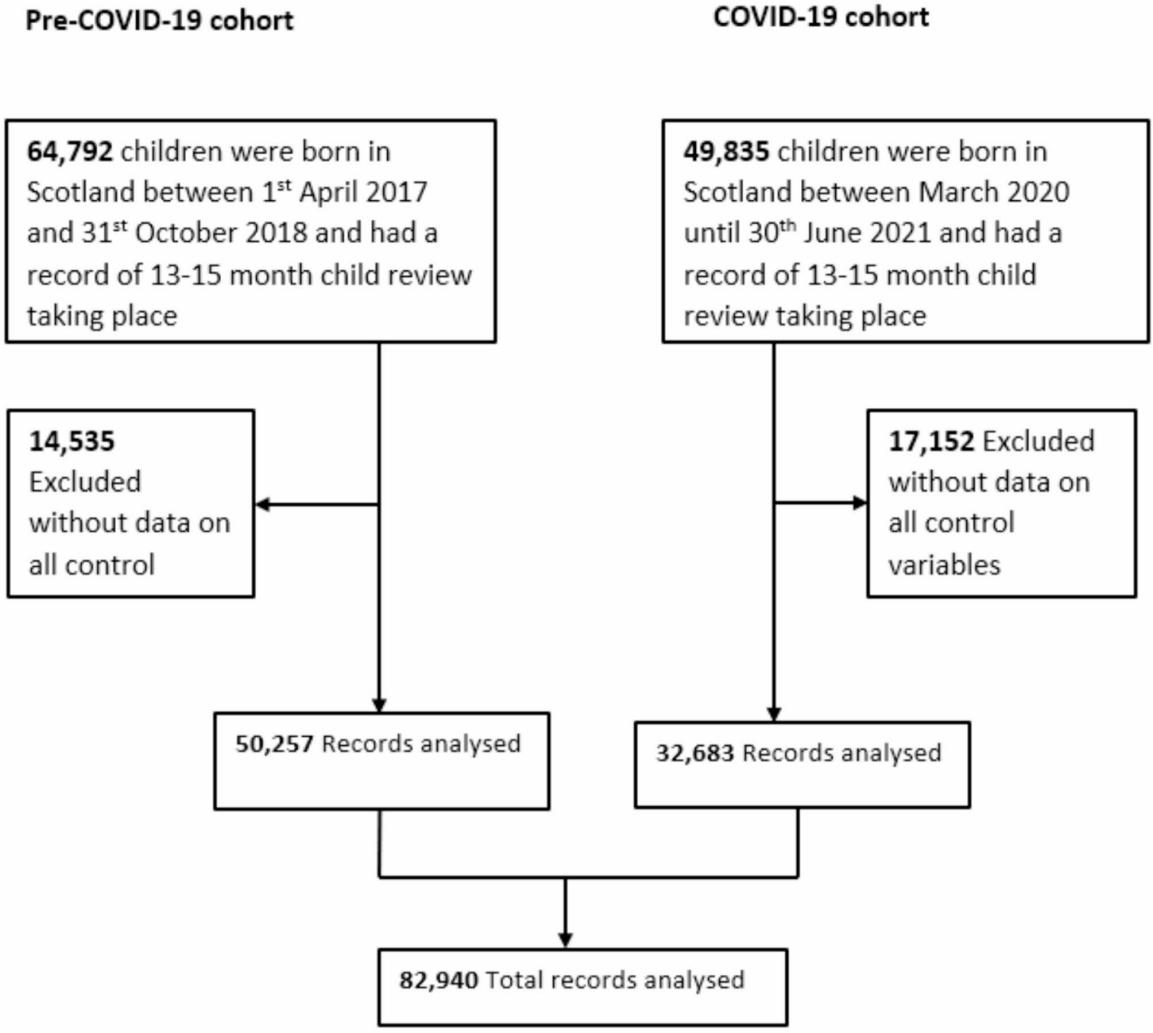
Participants flow chart (Children born between 1st April 2017 and 30th June 2021 in Scotland)

Our study utilised data collected as part of the COVID-19 Health Impact on Long-term Child Development in Scotland (CHILDS) study. The CHILDS study combines the population-based COVID-19 in Pregnancy in Scotland (COPS) dataset [23], Scotland’s 13–15 month child health reviews [24], Scottish birth records [25, 26], and MH hospital admission records and psychiatric outpatient attendance records [27, 28]. These datasets were obtained for a national cohort of all individuals (*n* = 49,835) who had live births from 1st March 2020 until 30th June 2021 in Scotland. We only included participants with complete matched child-mother data with outcome, predictors, and covariates (*n* = 32,683). We also included all live births in Scotland between 1st April 2017 and 31st October 2018 (*n* = 64,792) with complete matched child-mother data with outcome, predictors, and covariates. The start date for the pre-covid cohort was based on when the ASQ-3, 13–15 month health reviews were widely in use in Scotland [29], while the end date of October 2018 ensured that all children in this cohort had their 13–15 month child health review before the COVID-19 pandemic. However, we only included participants with matched child-mother data (*n* = 50,257) to compare the effects of the pandemic to a pre-pandemic historical cohort. Our sample for both cohorts comprised 82,940 participants, as shown in Fig. 1.

### Measures

#### Outcome variable

Outcomes were the children’s developmental outcomes measured by Ages and Stages Questionnaire (ASQ-3) scores [30] and developmental concerns identified by health visitors at the 13-to-15-month review. The reviews are routine visits offered to all children in Scotland and delivered by health visitors. The ASQ-3 scores are continuous variables that indicate the screening scores across five domains (personal-social, problem-solving, gross motor, fine motor, and communication) [29]. The ASQ-3 items are scored in one of three ways: ‘yes’ (equivalent to 10 points) if the child can perform the activity, ‘sometimes’ (equivalent to 5 points) if the child attempts but fails, but the primary caregiver believes the child can perform the activity occasionally, and ‘no’ (equivalent to 0 points) if the child is unable to perform the activity. The responses to each domain’s six questions are added together to obtain a score for each domain, with scores for each domain ranging from 0 to 60. A higher score indicates more positive outcomes for children. To calculate the overall ASQ-3 score, the total score in each domain is added together, with the total score ranging from 0 to 300 [30].

We used developmental concerns as the outcome variable to examine whether there is an association between maternal MH during pregnancy and child developmental concerns identified by health visitors during early child health reviews in both cohorts (pre-COVID-19 and COVID-19). Developmental concerns were defined as cases where a concern was identified in at least one developmental domain (personal-social, emotional/behavioural, speech, language and communication, fine motor skills, gross motor skills, vision, hearing and problem-solving) [31]. Health visitors make judgements on whether there is a concern based on the following: (a) elicitation of parental concerns, (b) a structured observation of the child, and/or (c) the ASQ-3 scores. This binary variable indicates whether any developmental concerns were identified by health visitors during each child’s 13–15 months health review (coded: 0 = no developmental concerns identified, 1 = at least one developmental concern identified).

#### Predictor variables

The predictor was measured by birth during the pandemic and maternal MH (history of MH hospital admission and psychiatric outpatient attendance records). These were binary variables with information on mothers who gave birth during the pandemic (1st March 2020 until 30th June 2021) and maternal MH. A history of MH hospital admission is recorded in the MH inpatient and day-case hospital admission data (SMR04) and was coded as a binary variable (coded 0 = no history of admissions; 1 = history of admissions, i.e. one or more recorded admissions). The psychiatric outpatient attendance record was obtained from the outpatient data (SMR00), specifically with G codes indicating that they attended the outpatient psychiatric clinic. It was coded as a binary variable (coded 0 = no; 1 = yes, i.e., new MH case or follow-up)—data source: psychiatric outpatient attendance data (SMR00) [28].

#### Control variables

Our model adjusted for the following covariates through Inverse Probability of Treatment Weighting (IPTW) [32]: maternal age, child’s sex, maternal smoking, area-based deprivation, and preterm birth. Maternal age was coded as an ordinal variable (coded: 0 = ≤ 19, 1 = 20–35, 3 = ≥ 36, i.e. in line with the ‘optimal’ (20–35) and ‘suboptimal’ (≤ 19/≥36) based on maternal age categorisations for neurodevelopment identified by previous research [33]. Maternal smoking status from the health records was coded as a categorical variable showing whether the mother was a smoker, ex-smoker or has no history of smoking (coded: 0 = never smoker, 1 = ex-smoker, 2 = current smoker). Area-based deprivation data were obtained from the Scottish Index of Deprivation (SIMD) quintiles and coded as a categorical variable (coded as 1 = least deprived, 2 = less deprived, 3 = medium deprived, 4 = more deprived, 5 = most deprived) [34]. Preterm birth was defined as any birth before 37 weeks completed weeks of gestation and was a binary variable (coded as 0 = no and 1 = yes).” These covariates were obtained from the maternity inpatient and day case records (SMR02) [28], including antenatal, delivery and postnatal data.

### Data analysis

The coverage of the dataset presented in this study is nationwide with diverse socioeconomic status; therefore, it necessitates creating a comparable sample prior to our analysis. To create a balanced pseudo-sample regarding covariates across the cohorts of comparable individuals, we generated weights for each participant based on the covariates. To generate the weights, we used a logistic regression model to estimate the propensity scores of each participant in each group (pre-COVID-19 and during COVID-19 cohorts) for our first and second models as the probability of having MH difficulties [35–37]. We included all covariates (SIMD, child sex, mother’s age, smoking status and preterm) as they could confound the relationship between maternal MH and children’s developmental outcomes [38]. Notably, all our covariates were associated with the predictor and outcome variables, unnecessarily limiting the opportunity to increase the variance [39]. Therefore, shared variance between the covariates and both the predictor and outcome variables might reduce the unique contribution of the predictor. Consequently, this might affect the observation of additional effects of maternal MH on developmental outcomes, potentially underestimating the strength of the relationship and, therefore, necessitating the use of Inverse Probability of Treatment Weights (IPTW).

We used the propensity scores to calculate each participant’s Inverse Probability of Treatment Weights (IPTW). Weights were calculated as a 1/propensity score for mothers with MH and 1/(1 - propensity score) for mothers without MH [32]. Therefore, subsequent inclusion of the weights in our model could render being assigned to the group as either having MH difficulties (having a history of MH hospital admission/psychiatric outpatient attendance) or not having MH difficulties independent of the variables included as covariates in the propensity score model [32]. Through this, participants with MH difficulties with a lower probability of having MH difficulties (and those without MH difficulties with a higher likelihood of having MH difficulties) receive higher weights. Therefore, their relative influence on the comparison is increased.

We used three models to estimate the effect of the COVID-19 pandemic on the association between maternal MH and children’s developmental outcomes. In the first model, we used a linear regression model with weighted data for the *pre-COVID-19* cohort to estimate the association between MH and developmental outcomes. In the second model, we used a linear regression model with weighted data for the *during-COVID-19* cohort to estimate the association between MH and developmental outcomes during COV. In the third model, weighting was done across the two cohorts (*pre-COVID-19* and *COV*) to get weights for the merged dataset. We then used a linear regression model with weighted merged data to estimate the association between MH and developmental outcomes and the effect of the interaction between being born during the COVID-19 pandemic and maternal MH. In both models, the outcome variable was child developmental outcome (*Y*), and maternal MH was the predictor variable. In addition, we included all covariates in all three outcome models for robustness.

We used standardized scores for the outcome variables in all the models and then reported the coefficients and standard errors of the outcome models as this could communicate the practical significance of our results [39]. Analyses were conducted using the following R packages; “*twang”* to estimate propensity scores [40], the “*ipw”* for weighting the dataset [41] and “*cobalt”* to calculate the standardised difference between the weighted and unweighted datasets for the covariates balance [42] within Scotland’s National Safe Haven.

### Sensitivity analysis

Noting the potential confounding effects of gestational age, we conducted a sensitivity analysis, repeating the same model in the primary analysis with participants with full-term babies, excluding the preterm.

## Results

### Demographic characteristics

The population in this study was relatively similarly spread across the five levels of SIMD in both cohorts; most deprived (pre-COVID cohort 20%, COVID cohort 18%), more deprived (pre-COVID cohort 21%, COVID cohort 21%), medium deprived (pre-COVID cohort 20%, COVID cohort 20%), less deprived (pre-COVID cohort 22%, COVID cohort 23%), and least deprived (pre-COVID cohort 17%, COVID cohort 17%). Of the 82,940 participants, 39.4% were born during COV. In terms of mothers’ MH, 1.8% (pre-COVID cohort 1.9%, COVID cohort 1.6%) had a history of MH hospital admission. Regarding children’s development, the mean total ASQ-3 score for children aged between 13 and 15 months for the pre-COVID cohort was 248.50 (SD = 40.63), and for the COVID cohort was 248.23 (SD = 41.91). There were slightly more males in both cohorts than female children. In addition, the mean ASQ-3 score was slightly higher in the pre-COVID-19 cohort than in the children born during COVID-19, as shown in Table 1.

**Table 1:** Demographic characteristics of participants (Children born between 1st April 2017 and 30th June 2021 in Scotland)

	Pre-COVID cohort; born between 1st April 2017 and 31st October 2018 (*N* = 50,257)	COVID cohort; born between 1st March 2020 and 30th June 2021) (*N* = 32,683)	*P* values
**Outcome**			
* Mean Total ASQ scores (SD)*	248.50(40.63)	*248.23(41.91)*	0.37
* Mean problem solving (SD)*	49.21(10.60)	*49.16(10.91)*	0.48
* Mean personal social (SD)*	51.12(9.76)	*51.34(9.75)*	0.002**
* Mean communication (SD)*	47.57(11.92)	*46.86(12.43)*	< 0.001 ***
* Mean fine motor (SD)*	49.83(10.32)	*50.03(10.49)*	0.007**
* Mean gross motor (SD)*	50.76(14.14)	*50.85(14.22)*	0.37
*Any developmental observation or concern identified between 6 weeks and 15 months reviews N(%)*			
* No*	42,546(84.66)	*26*,*831(82.09)*	
* Yes*	7,711(15.34)	*5*,*852(17.91)*	< 0.001 ***
**Predictors**			
History of MH admission N(%)			
* No*	49,298(98.09)	32,167(98.42)	
* Yes*	959(1.91)	516(1.58)	0.001**
MH outpatient attendance records N(%)			
* No*	41,947(83.46)	27,409(83.86)	
* Yes*	8,310(16.54)	5,274(16.14)	0.13
**Control variables**			
SIMD quintile *N*(%)			
* 1(most deprived)*	9,801(19.50)	6,099(18.66)	
* 2(more deprived)*	10,676(21.24)	6,986(21.38)	
* 3(medium deprived)*	10,132(20.16)	6,680(20.44)	0.001**
* 4(less deprived)*	10,910(21.71)	7,500(22.95)	
* 5(least deprived)*	8,738(17.39)	5,418(16.58)	
Maternal age at the time of birth *N*(%)			
* < 20*	2,264(4.50)	1,217(3.72)	
* 20–35*	41,281(82.14)	26,739(81.81)	< 0.001 ***
* > 35*	6,712(13.36)	4,727(14.46)	
Sex of the child *N*(%)			
* Male*	25,960(51.65)	16,821(51.47)	
* Female*	24,297(48.35)	15,862(48.53)	0.6
Maternal prenatal smoking *N*(%)			
* No*	32,490(64.65)	21,410(65.51)	
* Ex-smoker*	10,415(20.72)	7,022(21.49)	< 0.001 ***
* Yes*	7,352(14.63)	4,251(13.00)	

### Covariate balance

**Fig. 2:**
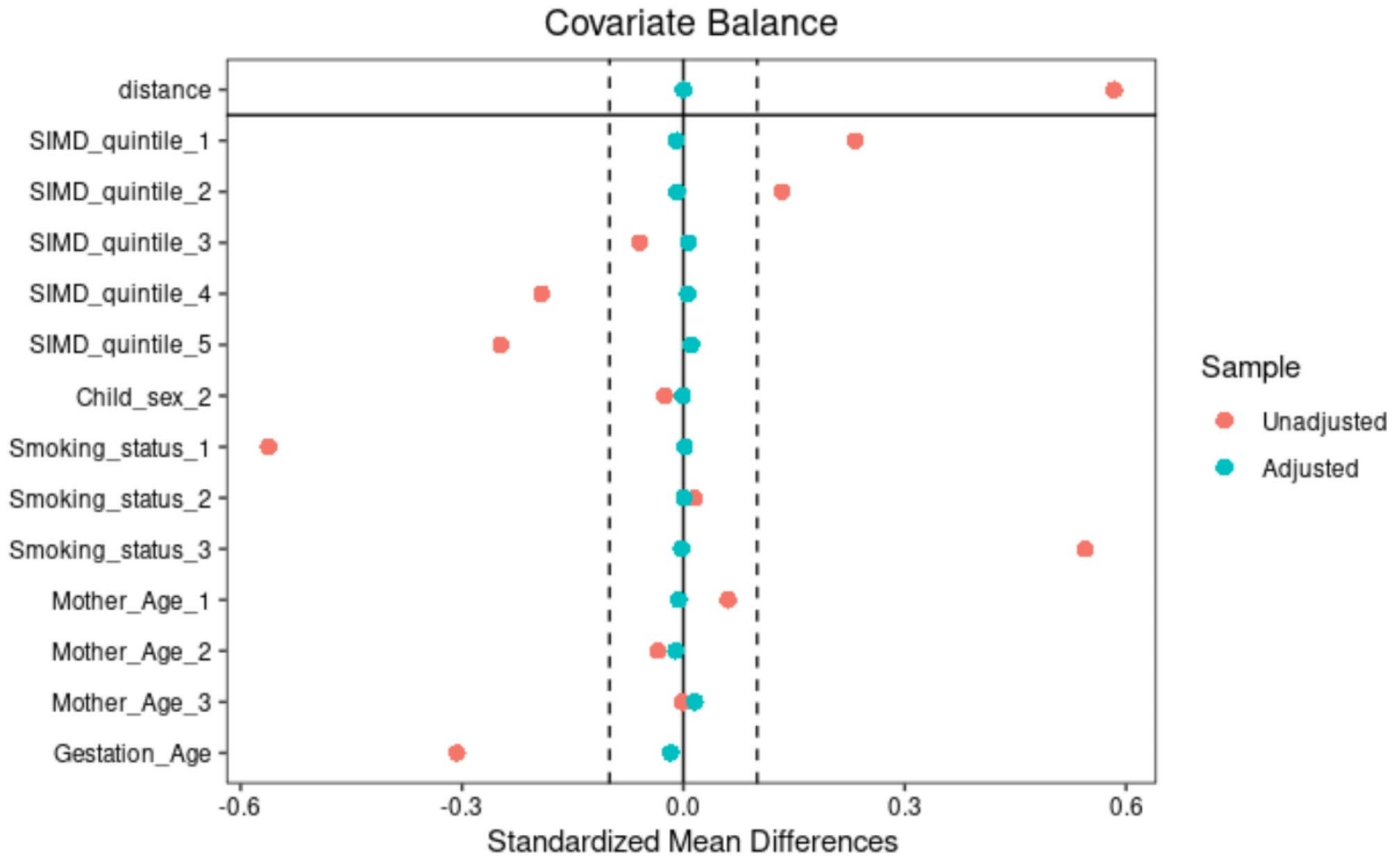
Covariate balance after weighting the dataset children born between 1st April 2017 and 30th June 2021 in Scotland

We checked the covariate balance in both the weighted and unweighted datasets using the standardised differences shown in Fig. 2. The result showed that all standardised differences were < 0.01.

### Association between maternal MH and children’s developmental outcomes

#### Pre-COVID-19 cohort

A linear regression model with weighted data for the pre-COVID-19 cohort was used to estimate the association between maternal MH and developmental outcomes during the pre-COVID-19 period, which showed that maternal MH was associated with children’s developmental outcomes. There was a significant negative association between maternal MH hospital admission and children’s ASQ-3 scores; those who had a history of MH hospital admission had children with 0.14 standard deviation lower than that of those without a history of MH hospital admission (ES= -0.140, 95% CI [-0.214, -0.065], *p* < 0.001***), as shown in Table 2. This indicates that the average ASQ-3 score for children of mothers with a history of MH hospital admission was 0.14 standard deviations lower than the average ASQ-3 score for children of mothers without a history of MH hospital admission.

**Table 2:** Association between maternal MH (Admission) and ASQ scores for both the COVID and pre-COVID cohorts (Children born between 1st April 2017 and 30th June 2021 in Scotland)

Pre-COVID cohort					
	Effect Size	Std. Error	95%CI Lower	95%CI Upper	Pr(>|t|)
Total ASQ scores	-0.140	0.038	-0.213	-0.065	< 0.001 ***
SIMD quintile					
* 1(most deprived)*	Reference				
* 2(more deprived)*	-0.007	0.016	-0.039	0.024	0.65
* 3(medium deprived)*	-0.014	0.015	-0.044	0.017	0.37
* 4(less deprived)*	-0.010	0.015	-0.039	0.020	0.52
* 5(least deprived)*	-0.039	0.015	-0.068	-0.009	0.009 **
Maternal age at the time of birth					
* < 20*	Reference				
* 20–35*	-0.178	0.025	-0.226	-0.130	< 0.001 ***
* > 35*	-0.270	0.027	-0.324	-0.217	< 0.001 ***
Sex of the child *N*(%)					
* Male*	Reference				
* Female*	0.187	0.009	0.171	0.204	< 0.001 ***
Maternal prenatal smoking					
* No*	Reference				
* Ex-smoker*	-0.016	0.012	-0.039	0.008	0.19
* Yes*	-0.063	0.022	-0.106	-0.019	0.005**
* Gestation age*	0.063	0.003	0.058	0.068	< 0.001 ***
**COVID cohort**					
Total ASQ scores	-0.210	0.053	-0.313	-0.108	< 0.001 ***

The findings on developmental outcomes, measured by developmental concerns identified by health visitors at scheduled health assessments, showed a positive association with MH hospital admission. That is, the odds of developmental concerns were 4.0% higher among children of parents with MH hospital admission than those without MH hospital admission (OR = 1.040, 95% CI [1.013, 1.066], *p* = 0.004**), as shown in Table 3.

**Table 3:** Association between maternal MH (Admission) and child developmental concerns for both the COVID and pre-COVID cohorts (Children born between 1st April 2017 and 30th June 2021 in Scotland)

Pre-COVID cohort						
		Estimate	Std. Error	95%CI Lower	95%CI Upper	Pr(>|t|)
Any developmental observation or concern identified between 6 weeks and 15 months reviews	No	Reference				
	Yes	1.040	1.014	1.013	1.066	0.004 **
SIMD quintile						
* 1(most deprived)*		Reference				
* 2(more deprived)*		-0.017	0.006	-0.028	-0.006	0.004 **
* 3(medium deprived)*		-0.018	0.006	-0.029	-0.007	0.001**
* 4(less deprived)*		-0.031	0.005	-0.042	-0.021	< 0.001 ***
* 5(least deprived)*		-0.044	0.005	-0.055	-0.034	< 0.001 ***
Maternal age at the time of birth						
* < 20*		Reference				
* 20–35*		-0.027	0.009	-0.045	-0.010	0.002 **
* > 35*		-0.015	0.010	-0.035	0.004	0.113
Sex of the child *N*(%)						
* Male*		Reference				
* Female*		-0.032	0.003	-0.038	-0.026	< 0.001 ***
Maternal prenatal smoking						
* No*		Reference				
* Ex-smoker*		0.006	0.004	-0.002	0.015	0.151
* Yes*		0.031	0.008	0.016	0.047	< 0.001 ***
* Gestation age*		-0.015	0.001	-0.017	-0.013	< 0.001 ***
**COVID cohort**						
Any developmental observation or concern identified between 6 weeks and 15 months reviews	No	Reference				
	Yes	1.067	1.020	1.028	1.107	< 0.001 ***

Similar trends were observed in the association between maternal MH assessed by psychiatric outpatient attendance and children’s developmental outcomes, as shown in Tables 4 and 5. MH assessed by psychiatric outpatient attendance was negatively associated with children’s ASQ-3 scores (ES= -0.089, 95% CI [-0.112, -0.065], *p* < 0.001***). In addition, MH assessed by psychiatric outpatient attendance was positively associated with developmental concerns (OR = 1.078, 95% CI [1.055, 1.102], *p* < 0.001***), as shown in Tables 4 and 5.

**Table 4:** Association between maternal MH outpatient and ASQ scores (Children born between 1st April 2017 and 30th June 2021 in Scotland)

Pre-COVID cohort													
	Effect Size			Std. Error		95%CI Lower		95%CI Upper			Pr(>|t|)		
Total ASQ scores	-0.089			0.012		-0.112		-0.065			< 0.001 ***		
**COVID cohort**													
Total ASQ scores	-0.141			0.016		-0.172		-0.109			< 0.001 ***		
**The interaction effect of being born during the COVID pandemic and maternal MH outpatient attendance on child developmental outcomes**													
Total ASQ scores *being born during COVID-19.			-0.066		0.020		-0.106		-0.027	< 0.001 ***			

**Table 5:** Association between maternal MH outpatient and child developmental concerns

Pre-COVID cohort													
			Estimate		Std. Error		95%CI Lower		95%CI Upper			Pr(>|t|)	
Any developmental observation or concern identified between 6 weeks and 15 months reviews	No		Reference										
	Yes		1.078		1.012		1.055		1.102			< 0.001 ***	
**COVID cohort**													
Any developmental observation or concern identified between 6 weeks and 15 months reviews	No		Reference										
	Yes		1.093		1.016		1.061		1.125			< 0.001 ***	
**The interaction effect of being born during the COVID pandemic and maternal MH outpatient attendance on child developmental outcomes**													
Any developmental observation or concern identified between 6 weeks and 15 months reviews *being born during COVID-19		No		Reference									
		Yes		1.018		1.020		-1.021		1.058	0.37		

#### During COVID-19 cohort

Similar findings were observed in the COVID-19 cohort. The findings showed a significant negative association between maternal MH hospital admission and children’s ASQ-3 scores; those who had a MH hospital admission had children with 0.21 standard deviation units lower than those without MH hospital admission (ES= -0.210, 95% CI [-0.313, -0.108], *p* < 0.001***) as shown in Table 2. In addition, the findings on developmental outcomes showed a positive association with MH hospital admission. That is, the odds of developmental concerns were 7.0% higher among children of parents with MH hospital admission than those without MH hospital admission (1.067 (95% CI [1.028, 1.107], *p* < 0.001***), as shown in Table 3. Similar trends were observed in the association between maternal MH assessed by psychiatric outpatient attendance and children’s developmental outcomes, as shown in Tables 4 and 5.

### Interaction effects of maternal MH hospital admission and being born during the COVID-19 pandemic on children’s developmental outcomes

In our third model, we used a linear regression model with weighted data for the combined cohorts (during the COVID-19 and pre-COVID-19 cohorts) to estimate the effect of the interaction between maternal MH and being born during the COVID-19 pandemic on children’s developmental outcomes. The findings showed mixed results on the association between being born during the COVID-19 pandemic and children’s developmental outcomes (developmental concerns: OR = 1.024, 95% CI [1.019, 1.029], *p* < 0.001***) and ASQ-3 scores (ES = 0.012, 95% CI [-0.002, 0.025], *p* = 0.08) for individuals without MH hospital admission as shown in Table 6.

**Table 6:** The association between maternal MH admission, being born during the COVID pandemic and child developmental outcomes (Children born between 1st April 2017 and 30th June 2021 in Scotland)

		Estimate	Std. Error	95%CI Lower	95%CI Upper	Pr(>|t|)
**Association between MH hospital admission and child developmental outcomes**						
Total ASQ scores		-0.130	0.038	-0.204	-0.056	< 0.001 ***
Any developmental observation or concern identified between 6 weeks and 15 months reviews	No	Reference				
	Yes	1.038	1.013	1.012	1.064	0.004 **
** Association between being born during the COVID pandemic and child developmental outcomes**						
Total ASQ scores		0.012	0.007	-0.002	0.025	0.08
Any developmental observation or concern identified between 6 weeks and 15 months reviews	No	Reference				
	Yes	1.024	1.003	1.019	1.029	< 0.001 ***
** The interaction effect of being born during the COVID pandemic and maternal MH hospital admission on child developmental outcomes**						
Total ASQ scores *being born during COVID-19.		-0.092	0.063	-0.218	0.034	0.151
Any developmental observation or concern identified between 6 weeks and 15 months reviews *being born during COVID-19	No	Reference				
	Yes	1.032	1.022	-1.012	1.076	0.150

The findings on the interaction effect of maternal MH and being born during the COVID-19 pandemic on children’s developmental outcomes showed mixed results. There was an interaction effect of maternal MH assessed by psychiatric outpatient attendance and being born during the COVID-19 pandemic on children’s ASQ-3 scores ES = 0.066, 95% CI [-0.106, -0.027], *p* < 0.001***). However, maternal MH assessed by hospital admission and the COVID-19 pandemic had no significant interaction on children’s developmental outcomes, as shown in Tables 4 and 6.

### Sensitivity analysis

Appendix A presents the sensitivity analysis results, which were repeated with participants with full-term babies, excluding the preterm. The results of the sensitivity analysis were broadly similar to the primary analysis.

## Discussion

This study examined the association between maternal MH and children’s developmental outcomes in pre-COVID-19 and COVID-19 cohorts. This study also examined the interaction effects of the COVID-19 pandemic and maternal MH on children’s developmental outcomes. Overall, the findings suggest that children of mothers with MH hospital admission were more likely to have reduced ASQ-3 scores and an increased likelihood of developmental concerns in both cohorts (pre-COVID-19 and COVID-19 cohorts). The findings also suggest that the interaction effect of being born during the COVID-19 pandemic and maternal MH hospital admission on children’s developmental outcomes was not statistically significant.

The link between MH difficulties and adverse child developmental outcomes was evident in both cohorts, as reflected in the ASQ-3 scores and the presence of developmental concerns. This association is consistent with other research that has established a correlation between maternal MH and adverse child developmental outcomes, particularly affecting cognitive and social-emotional development [43]. Moreover, a meta-analysis examining the impact of maternal perinatal depression and anxiety on children’s development from infancy to late adolescence corroborates our results [44]. These findings collectively suggest that maternal MH difficulties have a significant and lasting influence on children’s developmental trajectories from infancy through adolescence.

Our research indicated a small but significant association between the COVID-19 pandemic and children’s developmental outcomes. This observation aligns with studies conducted in China and the USA, which found that children born amidst the COVID-19 pandemic scored lower on the ASQ-3 than those born before it [22, 45]. Our findings also revealed a significant interaction between MH assessed by psychiatric outpatient attendance and being born during COVID-19 on children’s ASQ-3 scores. However, no interaction between COVID-19 and maternal MH was assessed by a history of hospital admission on child developmental outcomes. These findings suggest that being born during the COVID-19 pandemic and maternal MH influenced child development, and their combined presence could exacerbate this effect. However, the mixed findings on the interaction between maternal MH, assessed by psychiatric outpatient attendance and history of hospital admissions and being born during COVID-19 on children’s ASQ-3 scores were surprising and warrant further investigations.

Maternal MH difficulties have a known effect on children’s developmental outcomes [1–3]; however, limited studies have utilised large administrative population-level datasets to examine such associations and the potential interaction effects of the COVID-19 pandemic. The strength of the current approach is that it allows the inclusion of individuals who would not usually participate in research, as they rely on data routinely collected by healthcare and education professionals. Therefore, this study adds evidence on the effects of maternal MH difficulties on children’s developmental outcomes at a population level. The key strength of our research is the inclusion of a nationwide sample of matched mothers and children born between 1st April 2017 and 30th June 2021 in Scotland.

However, this study has several limitations. Since this is administrative data, it was not originally collected for this study. It might not fully represent the target population because of missing data from people not interacting with health services or inaccuracies in data linkage processes [46]. Notably, even though our model adjusted for covariates/confounders such as SIMD, gestational age, smoking and maternal age, other confounders could affect maternal MH and child developmental outcomes that were not accounted for in our model. Our study only examined developmental outcomes up to age 13–15 months; therefore, our findings only indicate potential future developmental delays [47]. In addition, the reduced face-to-face child health and maternal health assessment during the COVID-19 pandemic could affect the validity of measures used in this study for our predictors and outcome variables [37]. Despite these limitations, our study provides evidence of the link between maternal MH difficulties, being born during the COVID-19 pandemic, and children’s developmental outcomes; according to our knowledge, this is the largest population-level analysis. This is a significant contribution to the literature in regard to maternal MH difficulties during the COVID-19 pandemic and the potential worsening of their combined effects on children’s developmental outcomes.

## Conclusions

This study examined the interaction effects of the COVID-19 pandemic and maternal MH on children’s developmental outcomes. Our findings revealed that while the COVID-19 pandemic and maternal MH independently influenced child development, their combined presence, particularly MH assessed by psychiatric outpatient attendance, exacerbated this effect. Mothers with a history of severe MH and birth during the pandemic may each independently affect child development at 13–15 months to a modest degree. Further work is needed to understand whether dual exposure exerts long-term effects on children’s developmental outcomes.

## Electronic Supplementary Material

Below is the link to the electronic supplementary material.

Supplementary Material 1

## Data Availability

The administrative health datasets used for this study can be accessed by successful application to the NHS Scotland Public Benefit and Privacy Panel for Health and Social Care (HSC-PBPP).
