# Supplementary material for: Association between maternal mental health, the COVID-19 pandemic, and children’s developmental outcomes in Scotland
Source: Arch Public Health. 2025 Mar 27;83:82. doi: 10.1186/s13690-025-01572-w (PMC11948697; doi:10.1186/s13690-025-01572-w)
Supplement: Supplementary file 1 — Supplementary Material 1 [file 13690_2025_1572_MOESM1_ESM.docx]

Appendix A: The association between maternal MH, being born during the COVID pandemic and child developmental outcomes- Sensitivity analysis (Children born between 1^st^ April 2017 and 30th June 2021 in Scotland)

|  |  | Effect Size | Std. Error | 95%CI Lower | 95%CI Upper | Pr(>\|t\|) |
| --- | --- | --- | --- | --- | --- | --- |
| **Association between mental health and child developmental outcomes** | | | | | | |
| Total ASQ scores |  | -0.192 | 0.037 | -0.264 | -0.119 | **< 0.001 ***** |
| Any developmental observation or concern identified between 6 weeks and 15 months reviews | No | Reference |  |  |  |  |
|  | Yes | 1.060 | 1.013 | 1.034 | 1.087 | **< 0.001 ***** |
|  |  |  |  |  |  |  |
| **Association between being born during the COVID pandemic and child developmental outcomes** | | | | | | |
| Total ASQ scores |  | 0.005 | 0.008 | -0.010 | 0.020 | 0.51 |
| Any developmental observation or concern identified between 6 weeks and 15 months reviews | No | Reference |  |  |  |  |
|  | Yes | 1.03 | 1.003 | 1.021 | 1.032 | **< 0.001 ***** |
|  |  |  |  |  |  |  |
| **The interaction effect of being born during the COVID pandemic and maternal mental health on child developmental outcomes** | | | | | | |
| Total ASQ scores *being born during COVID-19. |  | -0.034 | 0.064 | -0.160 | 0.092 | 0.60 |
| Any developmental observation or concern identified between 6 weeks and 15 months reviews *being born during COVID-19 | No | Reference |  |  |  |  |
|  | Yes | 1.025 | 1.023 | -1.021 | 1.070 | 0.28 |
